# Is the “Family Glass Cabin” Useful to Safely Allow Inpatient–Caregiver Interaction in the COVID-19 Era? A Pilot Study on Severe Acquired Brain Injury

**DOI:** 10.3390/jcm11061623

**Published:** 2022-03-15

**Authors:** Rosaria De Luca, Carmela Rifici, Patrizia Pollicino, Sergio Parisi, Mirjam Bonanno, William Torregrossa, Donatella Ferrara, Angelo Caminiti, Marco Piccione, Rocco Salvatore Calabrò, Maria Cristina De Cola

**Affiliations:** Neurorehabilitation Unit, IRCCS Centro Neurolesi “Bonino Pulejo”, 98123 Messina, Italy; rosaria.deluca@irccsme.it (R.D.L.); carmela.rifici@irccsme.it (C.R.); patrizia.pollicino@irccsme.it (P.P.); sergio.parisi@irccsme.it (S.P.); mirjam.bonanno@irccsme.it (M.B.); william.torregrossa@irccsme.it (W.T.); donatella.ferrara@irccsme.it (D.F.); angelo.caminiti@irccsme.it (A.C.); marco.piccione@irccsme.it (M.P.); mariacristina.decola@irccsme.it (M.C.D.C.)

**Keywords:** Acquired Brain Injury, caregiver’s burden, COVID-19 pandemic, family glass cabin

## Abstract

Acquired Brain Injury (ABI) is a life-changing event that can have a devastating impact on all aspects of a person’s functioning. Patients with ABI present several behavioral problems that have worsened during the COVID-19 pandemic. This study aimed at investigating the role of a “Family Glass Cabin” (FGC) both in improving cognitive function and communicative abilities of people with ABI and in potentiating the mental health of their caregivers. Fifteen subjects affected by ABI and their caregivers were enrolled in this experimental study. Training was performed through the FGC and was based on either psychoeducational sessions for the caregivers or cognitive stimulations for the patients. The participants attended biweekly meetings for 12 consecutive weeks. Each participant was assessed by means of a complete psychometric and clinical battery, before (T0) and after (T1) the training. We found significant changes in all patients’ outcomes, including global cognitive function and communication abilities (*p* < 0.01), as well as an improvement in caregivers’ well-being. Our data suggest that the physical presence of the caregiver in the rehabilitation setting, using a safe setting such as the FGC, can be a valuable means to increase ABI patients’ functional recovery and reduce caregivers’ anxiety and emotional burden.

## 1. Introduction

Since January 2020, the COVID-19 pandemic has affected almost all countries and more than 380 million people around the world [1]. On 11 March 2020, the World Health Organization declared the COVID-19 outbreak a global pandemic. Governments started to operate in a context of radical uncertainty and had to face difficult health, economic, and social challenges. By spring 2020, more than half of the world’s population had experienced a lockdown with strong containment measures [2]. The disease, caused by the novel coronavirus SARS-CoV-2, usually manifests with fever (98%), cough (76%), dyspnea (55%), myalgia or fatigue (44%), headache (8%), hemoptysis (5%), and diarrhea (3%) [3]. In most severe cases, hypoxia and respiratory failure (61.1%) associated with arrhythmia (44.4%) and eventually multiple organ failure occur, requiring hospitalization in intensive care [4]. In particular, the acute manifestations of the virus appear to worsen in cases where the patient already has a previous chronic disease [5]. Although the majority of people infected with SARS-CoV-2 fully recover within a few weeks, a considerable number of patients still suffer from long-lasting problems similar to multi-organ damage in the acute phase of infection or experience symptoms continuously for a long time after recovery [6]. This illness, namely Long-COVID, is still poorly understood and affects survivors at all levels of disease severity, even younger adults, children, and those who were not hospitalized. The most common symptoms of Long COVID include fatigue, dyspnea, cognitive and mental impairments, chest and joint pains, palpitations, myalgia, smell and taste dysfunctions, headache, and gastrointestinal problems [6].

The COVID-19 disease, due to these disabling and diffusive characteristics, has also forced the Italian government to adopt the "lockdown" as a protective measure, especially in the hospital setting [7]. Lockdown, together with the fear of contagion, has caused not only physical but also emotional and psychological distress, and this long quarantine period was correlated with worse mental health [8]. Healthcare systems changed all over the world, and visits to inpatients were not permitted. Then, social distancing from loved ones increased a sense of uncertainty about their health status, with negative consequences on both patients and caregivers’ psychological well-being [9]. People with Acquired Brain Injury (ABI) suffer from cognitive, physical, and psycho-social problems and may experience anxiety, isolation, and apathy. Many individuals are unable to resume their premorbid roles within their family after ABI, and some become more reliant on loved ones for care [10,11]. The lack of social relationships is a common experience for many individuals with ABI, and reduced social integration often lasts for a long time, even over >10 years post-injury [12]. Notably, all these issues have been worsened by the pandemic. Indeed, since people with ABI often suffer from respiratory and cardiovascular comorbidities, the effect of SARS-CoV-2 infection in this patient population may be fatal. Moreover, to reduce virus infection spreading, caregivers’ visits to ABI inpatients were not permitted. Nonetheless, one of the few studies dealing with this important issue found that, in a cohort of 11 patients with ABI, COVID was unexpectedly moderate, caused at most mild respiratory distress, and did not result in fatalities [13].

An increase in psychosocial and behavioral problems was indeed shown in ABI patients. In fact, the Headway survey [14] indicated that 65% of their ABI respondents reported feeling isolated as a result of lockdown, and 60% reported that this had a negative impact on their mental health (including increased anxiety and fear of their future). Some rehabilitation services were organized to provide appointments via software, video conferencing, and online call platforms to re-establish contact between the patient and the medical team [15,16]. However, this presented inevitable challenges including the unreliability or inaccessibility of video conferencing software, the need to maintain patient confidentiality, and difficulties in using technology [17,18]. For patients suffering from ABI, the current pandemic situation represents an additional challenge in his/her care path [19]. The health emergency, in fact, has not allow canonical medical visits, as health workers have been employed in acute COVID-19 services. This has led to increased psycho-social distress, anxiety, isolation, and apathy [20]. Moreover, without the support offered by family and friends during hospitalization, these patients experienced extraordinary social isolation, which further increased stress, anxiety, and depression, with a negative impact on their motivation to participate in physiotherapy and rehabilitation [21]. Technology may help caregivers to break down barriers with loved ones in a meaningful way. In a previous study, it has been shown that on-line therapy can be a useful and complementary treatment, in addition to standard rehabilitation, to potentiate the global cognitive and functional recovery in ABI subjects, also reducing caregiver’s distress and burden [22]. Moreover, the role of caregivers is fundamental for a patient’s rehabilitation, especially when innovative devices have to be used to improve the cognitive function [23]. Nonetheless, the importance of the family environment in determining functional outcomes for adults with ABI has received less emphasis in the literature, especially during the COVID-19 pandemic [24]. Family members certainly have the potential to improve ABI outcomes, through interpersonal support and emotional contact [25], and their role as active members of the rehabilitation team has long been recognized. 

Our study aimed to investigate the role of a Family Glass Cabin (FGC) as a safe means to potentiate the global cognitive status and communicative abilities of people with ABI as well as to improve the mental health of their caregivers during the COVID-19 pandemics. 

## 2. Materials and Methods

### 2.1. Study Population

Fifteen subjects (11 males and 4 females) with a mean age of 56 years, affected by ABI (with a traumatic etiology in 30% of the cases and a vascular one in the 70%), and their caregivers (5 males and 10 females) who attended from October 2021 to December 2021 the Intensive Neurorehabilitation Care Unit of the IRCCS Centro Neurolesi “Bonino-Pulejo” (Messina, Italy) were enrolled in the study. A more detailed description of both patients’ and caregivers’ demographical conditions is reported in Table 1. 

The study was conducted according to the ethical policies and procedures approved by the local ethics committee (IRCCSME 45/21). All patients’ legal guardians gave their written informed consent to study participation and data publication.

Patients’ inclusion criteria were: (i) diagnosis of Acquired Brain Injury (vascular or traumatic etiology) in the post-acute phase (i.e., 3–6 months from the acute neurological event); (ii) age range between 18 and 75 years; (iii) Levels of Cognitive Functioning (LCF) score ≥ 2. Patients were excluded if they had severe cognitive/behavior and medical illness (i.e., cardiorespiratory instability) potentially interfering with the training.

### 2.2. Experimental Protocol

**Figure 1:**
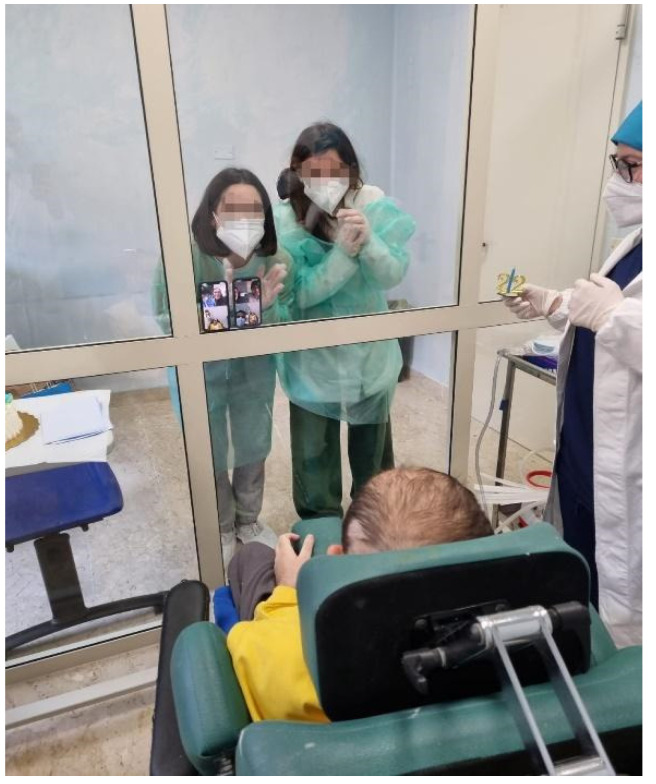
A young patient affected by ABI during the safe interaction with his caregivers (mum and sister).

The experimental training consisted of multidisciplinary meetings, in which the patients and the caregivers were separated by a glass cabin (namely, FGC) to allow for a safe interaction (see Figure 1).

The FGC was specifically built before the experiment started and was located in a room in front of the Neurorehabilitation ward. Thus, either a nurse or a physician was on-call if medical problems (including hypertension) occurred in patients attending the training. The participants attended biweekly meetings (every Tuesday and Thursday, in either the morning (h 12–14) or the afternoon (h 18–20)) for 12 consecutive weeks, in addition to standard neurorehabilitation. During the FGC sessions, a skilled psychiatric therapist and a psychologist held a specific focus group (including the two therapists, one or more caregivers, and the patients) to meet the needs of ABI patients and their caregivers. Before joining the group, despite the safety of the glass cabin, the caregivers were submitted to a molecular swab. The focus group is a research technique used to collect data through group interaction. The group comprises a small number of carefully selected people to identify and explore how people think and behave, posing why, what, and how questions.

The overall duration of an FGC session was about 80 min. The first phase of the focus group (lasting about 20 min) was mainly dedicated to the caregiver. The psychologist administered some specific therapeutic techniques: (i) a psychoeducational training, i.e., an evidence-based therapeutic intervention for patients and their loved ones that provides information and support to better understand and cope with illness; (ii) “defusing”, which consists of a short intervention organized through counseling groups to face the traumatic event; (iii) “debriefing” to reduce the emotional consequences of dramatic experiences, which is used in emergency psychology. The second phase (30 min) was characterized by a cognitive/sensory stimulation of the patients, using reality orientation therapy (ROT). This therapy was finalized to orient the patient in time and space, including also personal orientation. In the third phase (lasting further 30 min), emotion-focused therapy, a therapeutic approach based on the premise that emotions are key to identity, was applied. In this treatment model, the therapist and the patient collaborated in an active process aimed to process and recognize emotions, overcoming negative thoughts that may be associated with unhelpful or maladaptive emotions. Time for “free” interaction between the patients and the caregiver was also permitted (see Table 2), and caregivers’ emotions, thoughts, and feelings were discussed with the therapists.

Each participant was evaluated by a neurologist and a psychologist through the administration of a neuropsychological battery before (T0) and after (T1) the treatment.

### 2.3. Outcome Measures

The multimodal assessment consisted of: (i) Levels of Cognitive Functioning (LCF), one of the earlier developed scales used to assess cognitive functioning in post-coma patients [26]; (ii) Functional independence measure (FIM), an 18-item (13 motor [mot-FIM] and 5 cognitive [cogn-FIM]) assessment tool that explores an individual’s physical, psychological, and social function, used to determine the level of dependence of patients in daily life. This tool is used to assess a patient’s level of disability as well as changes in a patient’s status in response to rehabilitation or medical interventions [27]; (iii) Functional Communication Scale (FCS) to evaluate different items: motivation, collaboration, understanding, and language abilities, including verbal and non-verbal communication [28]. Moreover, specific behavioral scales were administered to the caregivers: (i) the Zung Self-Rating Anxiety Scale (SAS), a 20-item self-report assessment device built to measure anxiety levels, based on scoring in 4 groups of manifestations: cognitive, autonomic, motor, and central nervous system symptoms. Some questions are negatively worded to avoid the problem of set response [29]; (ii) the Zarit Burden Interview (ZBI-22), a popular caregiver self-report measure used by many aging agencies, consisting of items assessing caregivers’ burden [30]. Lastly, we evaluated the globally perceived quality of the use of the FGC by means of a structured interview focusing on specific items, including team participation, skills and reliability of the staff, usefulness of the service in the emotional management of the family member’s pathology, and whether the caregiver would recommend the use of the GFC.

### 2.4. Statistical Analysis

We performed a non-parametric analysis because of the reduced sample dimensionality and of the non-normal distribution of most target variables evaluated by means of the Shapiro–Wilk test. Thus, we used the Wilcoxon signed-rank test to compare the assessment scores between T0 and T1. Effect sizes were assessed by dividing the test statistic by the square root of the number of observations. The Chi-squared test was used to compare proportions. Continuous variables were expressed in median ± first-third quartile, whereas categorical variables in frequencies and percentages. Statistical analysis was performed using the 4.0.5 version of the open-source software R. A *p* < 0.05 was considered statistically significant.

## 3. Results

All participants completed the experimental training without any side effects. In particular, there was no need to call either nurses or physicians to manage hypotension and/or other medical problems potentially occurring during the training. No significant gender differences were found, in either the patients or the caregivers (Table 1).

Comparing the clinical and psychometric test scores between baseline and follow-up, we found significant changes in all patients’ outcomes: LCF (*p* < 0.01, d = −0.82), FIM (*p* < 0.01, d = −0.82), and FCS (*p* = 0.01, d = −0.67), as well as in caregivers’ outcomes: SAS (*p* < 0.001, d = 0.88), and ZBI-22 (*p* < 0.01, d = 0.87), as shown in Table 3.

Concerning the users’ satisfaction, around 92.34% of caregivers declared of perceiving the global quality of the use of the FGC as being from good to excellent. About 66.67% of caregivers perceived as excellent both team participation and skills and reliability of the staff (the remaining 33.33% evaluated them as good). The whole sample considered the FGC useful for the management of the family member’s pathology and would recommend its use.

## 4. Discussion

To the best of our knowledge, this is the first hospital-based experiment to investigate the psychometric and clinical outcomes of a special training using the “Family Glass Cabin” during the COVID-19 pandemic. Indeed, this promising tool may safely overcome social distance to allow a direct contact between people with ABI and their caregivers. It was observed that the FGC had positive effects on both stimulating functional recovery in the patients and reducing anxiety and burden in their caregivers. In particular, our data suggest that the real presence of a caregiver (although mediated by the FGC) can be useful to potentiate general communication and interpersonal abilities and improve the global cognitive status and sensory–motor outcomes of patients with ABI. Moreover, the FGC was also useful to provide not only the patients but also their caregivers with an emotional and social experience promoting the caregivers’ psychological well-being. Indeed, caregivers’ emotional burden and anxiety symptoms were significantly reduced at the end of the experiment. 

Family members of people affected by ABI have long been recognized as being significantly affected by the injury to their relatives. Research addresses the several difficulties experienced by a patient’s family, including changes in the physical abilities of people affected by ABI, potential changes in their personality and behavior, reduced social contact and community integration, as well as role and relationship changes, e.g., from partners/equals to providers of care/support [31,32]. Social distance is a serious limitation, but in the COVID era it becomes a limit that cannot be neglected. The impossibility to visit/be visited by their relatives due to the COVID-19 restrictions can cause serious behavioral problems in both patients and caregivers. In fact, the family’s ability to cope with stress can influence the quality of support they provide to their loved ones and, consequently, the extent of the recovery of ABI survivors [33,34,35]. Families vary in how they cope with the stress and burden of caregiving. Effective problem-solving can decrease anxiety and depression in caregivers [36] and may allow them to better cope with the demands of caregiving [37,38]. The pandemic has forced many caregivers of individuals with chronic or acute illnesses into a distant care-giving role, amplifying their anxiety and distress [39,40]. In particular, anxiety and post-traumatic stress may be exacerbated in caregivers who feel an additional caregiving burden during disasters [41]. COVID-19-related restrictions have allowed the systematic study of the degree to which social distancing also affects tactile experiences and mental health. In fact, it is undeniable that social touch has positive effects on social affiliation and stress alleviation [42]. Moreover, eye contact as well as other forms of non-verbal communication play an important role in human interactions. To overcome some issues related to the pandemic restrictions, telemedicine could be of some help [43,44,45]. Indeed, we previously used an on-line approach to allow a better interaction between patients with ABI and their caregivers during the first lockdown in Italy. According to our experience, carried out in an Intensive Neurorehabilitation Unit, the on-line therapy (using Skype) was effective to relieve caregivers’ distress and burden, with a positive impact also on the patient [22]. However, communicating online can introduce particular challenges in sustaining an emotional, social, and family relationship, especially if it lasts for a long time [39,46]. The lack of the physical presence of another person in the same room may make some people feel less emotionally intimate and less comforted when they are stressed. This is why, besides the positive outcomes, our previous study on the use of the “online therapy” [22] presented several problems, including (i) difficulties in communicating emotions and thoughts, (ii) a greater chance of miscommunication between ABI patients and their caregivers, and (iii) a higher risk of missing important non-verbal cues. Moreover, an unstable internet connection could further complicate productive communication. The awareness of these inspired the idea of using the FGC to reduce the social distance between ABI patients and their families, in the respect of the safety rules. Notably, we believe that the innovative use of the FGC could be advantageous to improve communication skills thanks to the activation of mirror neurons (MN). In fact, the direct observation of a caregiver’s movement can stimulate MN, promoting a better empathic interaction and social-cognitive processes [40]. The idea of how the MN mechanism helps us to understand others is called embodied simulation. According to Gallese [47], we have an immediate understanding of another person’s emotional and mental state by the automatic simulation of that person’s motor state in the MN of our own brain. Moreover, studies on the imitation of emotional facial expressions [48], as well as studies investigating additional social-cognitive processes—such as the Theory of Mind (ToM) and empathy—revealed activations in regions of the face-processing network and the MNS [49,50]. Then, this cue could be used to further potentiate cognitive and behavioral outcomes in patients with neurological disorders [51], including those with ABI (as in our study). Indeed, our experience suggests that the social and emotional presence of the caregivers can be considered a significant task-oriented stimulus (above all, their facial expressions) to potentiate the communicative abilities in these patients.

It is noteworthy that most caregivers rated the experiment as good/excellent and would recommend it in future settings, with a superior level of satisfaction, compared with our previous online services. In particular, the higher satisfaction was correlated with both the possibility of meeting their loved ones after a long time and the kindness/competences of the healthcare professionals.

Our study has some limitations. First, the lack of a control group receiving other kinds of support therapy may have biased the interpretation and generalization of the results. However, this was intended as a pilot study aimed at collecting evidence that could allow planning a future, confirmatory study on the clinical applicability of the FGC in real, large-scale rehab settings. Second, the sample size was very small, although it was complied with the inclusion criteria, and no formal statistical hypothesis was done a priori. Nonetheless, the prevalence of severe ABI is not so high, and evidence/research coming from this hospital population is necessary to advance our knowledge so to better manage these frail individuals. Finally, we lack a long-term follow-up and are unaware of whether the effects of this intervention may last over time. Further larger multicenter studies, with more homogenous samples and long-term follow-up, should be fostered in order to confirm these promising findings.

## 5. Conclusions

In conclusion, our data suggest that the FGC may be a valuable tool to promote functional recovery in ABI patients thanks to its potential to improve cognitive function and social skills such as communication. Moreover, it may be useful in relieving caregivers’ distress and burden, with a consequent positive impact on the patient. This novel approach could be a complementary tool for the neurorehabilitation of frail individuals, such as patients affected by ABI, especially in periods when social distancing is fundamental to counteract contagion diffusion. 

## Figures and Tables

**Table 1:** Socio-demographic and clinical characteristics of the sample at baseline.

	All	Males	Females	*p*-Value
Patients	15	11 (73.33)	4 (26.67)	
Age (years)	55.87 (15.42)	53.45 (15.20)	62.50 (16.11)	0.37
Education				0.83
Elementary school	3 (20.00)	2 (18.18)	1 (25.00)	
Middle school	7 (46.67)	5 (45.46)	2 (50.00)	
High school	3 (20.00)	2 (18.18)	1 (25.00)	
University	2 (13.33)	2 (18.18)	0 (0.00)	
Etiology				0.95
Vascular	13 (86.67)	9 (81.82)	4 (100.00)	
Traumatic	2 (13.33)	2 (18.18)	0 (0.00)	
Caregivers	15	5 (33.33)	10 (66.67)	
Age (years)	48.67 (9.54)	45.40 (11.55)	50.30 (8.58)	0.43
Education				0.05
Elementary school	1 (6.67)	1 (20.00)	0 (0.00)	
Middle school	6 (40.00)	0 (0.00)	6 (60.00)	
High school	8 (53.33)	4 (80.00)	4 (40.00)	
University	0 (0.00)	0 (0.00)	0 (0.00)	
Relationship with patient				0.06
Spouse	6 (40.00)	0 (0.00)	6 (60.00)	
Parents	3 (20.00)	1 (20.00)	2 (20.00)	
Son/Daughter	3 (20.00)	1 (20.00)	2 (20.00)	
Brother/Sister	1 (6.67)	1 (20.00)	0 (0.00)	
Other	2 (13.33)	2 (40.00)	0 (0.00)	

Quantitative data are in mean (standard deviation), qualitative data in frequencies (percentages).

**Table 2:** Phases of a typical Family Glass Cabin session.

Family Glass Session	ABI-Domain Intervention	CaregiverDomain Intervention	Focus Group Phases	Session Time
Face-to-Face Glasses Caregiver Meeting Glass Cabin Modality	Global Cognition Sensory–Motor Functioning Communicative Abilities Vigilance	Anxiety and depression symptoms Emotional Burden _______	First Phase Caregiver Education 20 min Second Phase Sensory Stimulation 30 min Third Phase Emotional Training 30 min	Caregiver 20 min 60 min depending on patient’s clinical condition and fatigue degree
	Emotions ________ ROT Emotion-Focused Therapy	Psycho-educational Defusing Debriefing		

**Table 3:** Statistical comparisons of clinical scores between baseline (T0) and follow-up (T1).

	Assessment	Baseline	Follow-Up	*p*-Value	ES
PATIENTS	LCF	5.0 (3.0–6.5)	7.0 (5.5–8.0)	**<0.01**	−0.819
	FIM	26.0 (18.0–52.0)	40.0 (21.5–65.0)	**<0.01**	−0.817
	FCS	31.0 (23.5–41.0)	41.0 (23.5–67.0)	**0.014**	−0.668
CAREGIVERS	SAS	59.0 (49.5–74.0)	45.0 (39.5–59.5)	**<0.001**	0.881
	ZBI-22	56.0 (53.5–62.0)	49.0 (43.0–51.0)	**<0.01**	0.874

Scores are in median (first-third quartile); significant differences are in bold. Legend: ES = Effect Size; LCF (Levels of Cognitive Functioning); FIM (Functional Independence Measure); FCS (Functional Communication Scale); SAS (Zung Self-Rating Anxiety Scale); ZBI-22 (Zarit Burden Interview).

## Data Availability

Data will be available on demand, from the corresponding author.
